# Behavioral Change Intervention to Promote a Healthier Postpartum Lifestyle: Mixed Methods Pilot Study

**DOI:** 10.2196/69391

**Published:** 2025-10-22

**Authors:** Pernille Kjærgaard Christiansen, Trine Kjær, Christina Anne Vinter, Mette Juel Rothmann, Mette Maria Skjøth, Eva Draborg

**Affiliations:** 1 Applied Business Research, UCL University College, Seebladsgade 1, Odense, 5000, Denmark, 45 26521787; 2 OPEN, Clinical Institute, University of Southern Denmark, Odense, Denmark; 3 Center for Innovative Medical Technology (CIMT), Odense, Denmark; 4 Department of Public Health, Danish Centre for Health Economics, University of Southern Denmark, Odense, Denmark; 5 Steno Diabetes Center, Odense, Denmark; 6 Department of Clinical Research, University of Southern Denmark, Odense, Denmark; 7 Department of Gynecology and Obstetrics, Odense University Hospital, Odense, Denmark; 8 Department of Dermatology and Allergy, Odense University Hospital, Odense, Denmark

**Keywords:** diet, exercise, health technology, motherhood, mixed methods, postpartum health

## Abstract

**Background:**

Research has shown that many mothers lack tools needed to motivate and support themselves in a healthy lifestyle after giving birth. A mobile health app (mHealth) has the potential to become a tool to accommodate this need. Accordingly, Healthy Together—a module in a general mHealth app, My Hospital including Podcasts, weight tracking, and exercise videos—was developed.

**Objective:**

The aim was to assess mothers’ use of the behavioral intervention, Healthy Together, which aims to support new mothers in a healthier lifestyle. Further, it evaluates mothers’ experiences and attitudes when using the intervention.

**Methods:**

A mixed method pilot study was conducted, and 34 women were included. From 3 weeks to 6 months postpartum, the women were granted access to Healthy Together. App activity was registered during the intervention period. All of the women received a questionnaire at the end of the intervention period; of these, 28 responded. In addition, 18 women participated in an online, semi-structured interview.

**Results:**

On average, each invited participant accessed the module 37 times. Push notifications and podcasts were used by 65% (n=34), and thus the content used the most. One-third found push notifications motivating. Half used exercise videos, while slightly fewer utilized weight tracking. A total of 70% (n=28) of those who answered the questionnaire had used Healthy Together. About half of the users reported that the intervention had a positive influence on their health status, and 70% (n=20) of the users stated they would recommend Healthy Together to others. The mean age was 29.8 years for users and 32 years for non-users. Pre-pregnancy body mass index averaged 25 and 24.8, respectively, increasing to 25.6 and 25.7 at 6 months postpartum. The body mass index difference was 0.6 for users and 0.9 for non-users, corresponding to total increases of 2.4 and 3.6, respectively. By gradually introducing the mothers to new content, the mothers could more easily digest the information. Podcasts were, in general, the preferred information channel. Weight tracking reminders motivated some, while they had the opposite effect on others. The users seemed to be those who were more physically active and had a healthier diet prior to their pregnancy, compared to non-users. About one-third of the users experienced technical problems.

**Conclusions:**

This study demonstrates that Healthy Together potentially is a feasible tool to assist women in improving their postpartum lifestyle. Further research with a larger sample is needed.

## Introduction

Pregnancy is a strong contributor to women gaining weight [1]. According to a study by Rogozińska et al [2], more than one-third of women exceed the Institute of Medicine’s (IOM) recommendations regarding weight gain during pregnancy. These women have an increased risk of not returning to their pre-pregnancy weights [3,4]. Following Villamor and Cnattingus [5], postpartum weight retention is associated with maternal obesity and adverse birth outcomes in subsequent pregnancies. As shown by Robinson [6] and Muttarek [7], being overweight has become so common that some overweight or obese people do not recognize themselves as being overweight, and health-related risks as the result of having a high BMI are often underestimated. Studies of the postpartum period show that women generally feel unprepared for the physical changes that come with pregnancy and birth and lack support of how to cope with these changes [8-11]. Although the educational preparations that women receive mainly focus on breastfeeding and infant care, there is not much focus on lifestyle and the postpartum woman’s physical and emotional functioning [10,12]. Lack of knowledge about the physical changes that birth causes can make mothers afraid of exercising after birth, causing them to be inactive [12]. In addition, focus on the baby can cause mothers to give their own lifestyles less priority [12]. As pointed out by Walker et al [13], and our previous studies [12,14,15], there seems to be an unmet need for lifestyle support among women in the postpartum period. Mothers need better knowledge about the importance of a healthy postpartum lifestyle, as well as guidance on how to maintain a healthy lifestyle.

There is evidence that health care professionals (HCPs)—in particular, hospital doctors—have a high rate of being trusted [16]. Therefore, there is the potential to make information and guidance on postpartum lifestyles available by having relevant HCPs deliver the information. Most mothers of childbearing age use information technology daily [17], giving it the potential as a tool to deliver important information and guidance. Mobile health (mHealth) apps have increasingly been used to promote health, and the number of mHealth apps is expected to increase even further in the years to come [18,19]. However, as Eysenbach [20] has pointed out, it is important that the app is reliable and established for its purpose, as technical issues can have an impact on whether a person will use an app.

The hospitals in the Region of Southern Denmark use the mHealth app called My Hospital [21]. Each department can have one or more modules in the app. The app is free of charge and follows European GDPR standards [22]. To support women in maintaining a healthy postpartum lifestyle, Healthy Together was developed as a module within My Hospital [23] and pilot tested at the Gynecological Department at Odense University Hospital.

The aim of this paper was to examine postpartum women’s experiences with, and attitudes toward, the use of Healthy Together, along with their frequency of use of the module, during the initial intervention period. In this paper, we examine whether Healthy Together enabled change in lifestyle behaviors in terms of diet and exercise and further identify possible barriers and facilitators to the module’s use, including preferences for individual components: push notifications, exercise videos, weight tracking, and podcasts. A mixed methods approach was used to gather more comprehensive data.

## Methods

### Setting and Participants

The pilot study on implementation of Healthy Together was carried out in the Region of Southern Denmark between June and December 2020 at the Department of Gynecology and Obstetrics at Odense University Hospital. It included women who had just given birth and were over 18 years old, Danish-speaking, and agreed to participate. Exclusion criteria were mothers from vulnerable families who are cared for by a family center, which provides support for families that face challenges related to abuse, distress, and other social problems. The study did not consider any medical or surgical interventions, such as cesarean or medication. Breastfeeding was also not considered.

### Healthy Together Module

Pregnant women in the Region of Southern Denmark are encouraged to use the My Hospital app through which they have access to all their appointments with the hospital and midwives, as well as leaflets [24]. Through the app, they can also communicate with a midwife. A new module, Healthy Together, was developed and added to the existing content [23]. The module’s aim is to support new mothers to a healthy lifestyle that focuses on diet and exercise as delivered through podcasts, weekly push notifications, exercise videos, and self-monitored weight tracking. A healthy lifestyle is conceptualized as the mothers’ self-assessment of the beneficial influence of the intervention on their individual health.

Following Kushniruk and Nøhr [25], the module was co-created with HCPs, mothers (intended users), and IT experts to define an intervention that met the women’s actual needs [24] and was usable. Mothers who participated in developing the intervention expressed a wish for podcasts as the preferred information channel. They already listened to podcasts and found it convenient that they could listen while keeping their hands and eyes free [24]. Their view is in accordance with the increasing use of podcasts in Denmark, and in particular, among young people, with 60% of 20‐ to 39-year-olds listening to podcasts in 2020 [26]. The mothers wished for exercise videos as they allowed them to exercise from home when the time would best suit them [24]. In a study by Laframboise and colleagues, virtual exercise videos have been used, with promising results, to reduce diastasis recti abdominis in postpartum women [27]. Previous studies have found push notifications to be effective for adhering to behavioral change interventions [28,29]. Push notifications were therefore proposed to remind the mothers about the presence of Healthy Together, while allowing them to gradually be introduced to the content in the module [24]. Finally, self-monitoring of weight gain/loss was added, as the mothers wanted a way to follow their own weight development. Studies show that regularly self-monitoring can improve weight loss outcomes [30,31]. Research also shows that new technologies, such as smartphone apps, may support adherence to self-monitoring [32]. Healthy Together sent the women weekly push notifications containing advice related to a healthy postpartum lifestyle and information about where in the module they could find further information on the topic. The module contained 6 podcasts and 12 exercise videos. The exercise videos acknowledge that women are advised to delay physical activity for 4-6 weeks postpartum, with an extended recommendation of 6-8 weeks for those who have undergone a cesarean section. The exercises provided were precisely the same as those utilized in in-person postpartum exercise classes.

From 8 weeks postpartum, the women also had the opportunity to track their own weight by self-monitoring it on a regular basis and inputting the information under My data in the module. Every third week, they received a reminder about weight tracking in the module. The women could download the app on their own smartphones. They could also access the content in the app on a tablet or a computer.

### Data

#### Overview

Data were gathered in a cross-sectional study. A mixed method containing information from data on actual use of the app, a questionnaire, and individual semi-structured interviews was used for the analysis following Creswell and Clark [33]. Hence, a mixed method concurrent design was applied. Data from the semi-structured interviews were triangulated with some of the questions from the questionnaire. From June 5 to July 10, 2020, a medical doctor and two nurses at the maternity wards at Odense University Hospital consecutively invited 40 new mothers to receive further information about the pilot study prior to their hospital discharge after the birth. Due to COVID-19, the hospital only had resources to recruit 40 new mothers. All women gave their consent to be contacted and to receive more information. Between 2 and 3 weeks postpartum, the first author contacted the women by phone or email. They were informed about the study and were asked to participate.

#### Extraction of Data From Healthy Together

From the app, we gathered summary data on the number of times each content page in the module was visited over a period of 6 months. Data were retrieved once all the women finished the intervention period. The statistics do not contain individual-level information but provide information on the total number of times that all participants visited during the intervention period.

#### Questionnaire

The questionnaire consisted of 33 questions about the women’s experiences using Healthy Together and its components, their general use of technology, sociodemographics (income, education, employment, marital status, and number of children in the household), subjective well-being and health behaviors (weight, weight gained during pregnancy, diet, exercise, and general mode). The questions about well-being are from the WHO-5 well-being index, using 6-point Likert scales [34]. The respondents were divided into users and non-users and compared. Respondents’ BMI and deviations from recommended weight gain according to the IOM were calculated based on self-reported information about height and weight. A translated version of the full questionnaire can be found in Multimedia Appendix 1. Data were gathered and stored in My Hospital, from which they were retrieved.

The questionnaire was pretested on 6 postpartum women, resulting in minor modifications. At 6 months postpartum, each woman received the questionnaire. Respondents participated in a lottery for a 500 Danish Krones (about US $75) voucher to shop for baby equipment. Reminders were sent after a week, and again after 2 weeks to those who had not previously replied.

#### Semi-Structured Interviews

A semi-structured interview guide with open-ended questions was developed to gather information about the women’s experiences using Healthy Together [35]. The questions included whether they used the module, what their experiences were from using it, and if it had an impact on their lifestyles. The women were also asked if they had any suggestions for improvements and if they would recommend the app to others. The interview guide was pretested on a postpartum woman and is presented in Multimedia Appendix 2.

Due to restrictions related to the COVID-19 pandemic, the interviews were conducted by telephone or Zoom. All participants were invited to participate in an interview. When invited by phone, those who said they did not use or only scarcely used Healthy Together were asked if there were any specific reasons for this. Interview statements used in this publication were translated from Danish to English. The first author, who was a female PhD student, conducted and transcribed all interviews. The participants were informed that she was a PhD student at the University of Southern Denmark, who took part in the study.

#### Methodological Orientation

The study consisted of iterations, where each iteration gave new insight into the next iteration, in which the hermeneutic cycle was applied. The questionnaire was used to gather information on the women’s attitudes toward the intervention and their experiences of using it. For this evaluation, a positivistic approach was applied [36].

### Analysis

Data from the app were available on the aggregate level and therefore could not be merged with the questionnaire data. Descriptive results from the questionnaire were reported by user status. Unfortunately, due to the limited sample size of the questionnaire data, the statistical testing of differences was not applicable and was therefore not performed.

Field notes were made, and the interviews were recorded on a dictaphone and transcribed in NVivo12 (version 12.6.1; Lumivero) and analyzed by the first author [37]. The interviews were about 30-45 minutes each. The interview data were analyzed using systematic text condensation (STC) [38,39]. An STC was made in 4 steps, where the first step was obtaining a total impression by reading the transcripts several times. In the second step, meaning units were identified. In the third step, overall themes were defined. Finally, in the fourth step, the content from each meaning unit was abstracted and synthesized.

### Ethical Considerations

The study complied with the Declaration of Helsinki and Danish Code of Conduct for Research Integrity [40]. The Danish Data Protection Agency (case no. 17/43487) approved the study, and it was submitted to the Scientific Ethics Committee for assessment. It was determined that the study did not require their approval, according to Danish legislation because no biological material or treatment was involved [41]. All women were given written and verbal information about the project. They were given an informed consent in which they were informed that their participation was voluntary, their data and statements would be anonymized, and they could withdraw their participation at any time. Participants did not receive any compensation.

## Results

### Patient Characteristics

**Figure 1.**
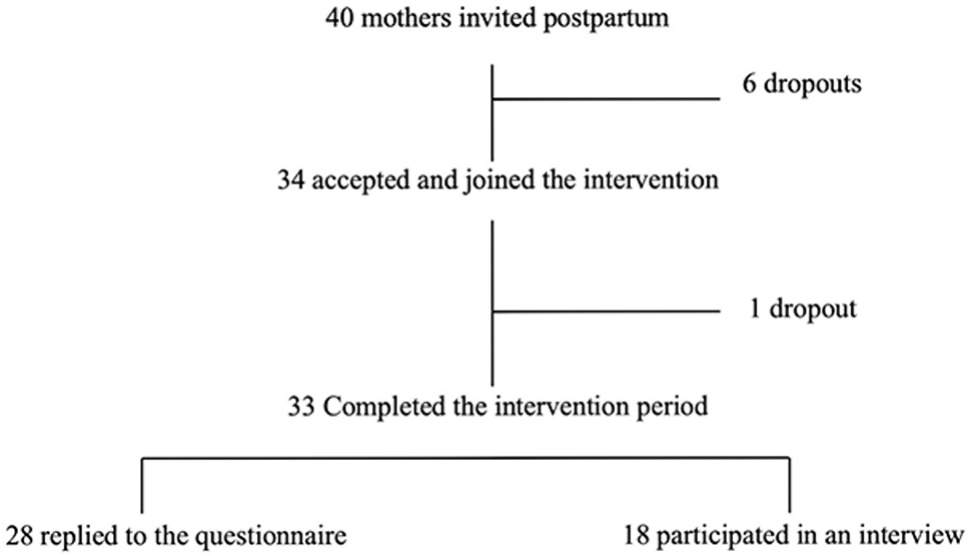
Flowchart of recruitment of postpartum women from the Region of Southern Denmark, who participated in the study Healthy Together from June to December 2020.

In total, 85% (n=34) of the 40 mothers invited to the study accepted to participate. The first author granted them access to the module. All of the women had access to the module between 3 weeks postpartum and 6 months postpartum. One participant dropped out after a few weeks, due to technical problems with the app. A total of 28 women replied to the questionnaire, and 18 participated in an online, semi-structured interview after the intervention period (some did both). Interviews were conducted between December 2020 and January 2021. A flowchart of the study is shown in Figure 1.

### Data on Use of Healthy Together

The complete use of the module during the intervention period is displayed in Table 1. There was a variation in the use of the different elements in the module, with push notifications being used the most and contact information the least. On average, each of the invited participants visited the module 37 times. As we are not able to contingent on user status, this number is likely to cover a large variation in use (from zero to high use).

**Table 1.** Use of the module Healthy Together by postpartum women in the Region of Southern Denmark, who participated in the study (users and non-users), retrieved from the app (June-December 2020; n=34).

Menus	Number of visits	Average visits per participant (n=34)	Average visits per week per participant (n=34)
Push notifications	586	17	0.7
Exercise videos	167	5	0.2
Podcasts	164	5	0.2
My data (weight tracking)	151	4.5	0.2
About	112	3.3	0.1
Contact information	65	2	0
Total	1264	37	—^a^

a not applicable.

### Questionnaire Findings

A total of 28 of the 33 participants (85%) responded to the questionnaire. The characteristics of the respondents at 6 months postpartum are shown in Table 2, grouped by user app status.

Overall, 70% (n=28) of the respondents stated they used Healthy Together. The respondents were between 23 and 40 years old at 6 months postpartum. The results seem to suggest some differences across user status (although not tested and therefore cannot be verified). The mean age of users was 29.8 years, whereas it was 32 years for non-users. Mean prepregnancy BMI for users and non-users at 6 months postpartum was 25 and 24.8, respectively. None of the respondents were underweight. During their pregnancies, half of the users exceeded the IOM’s weight gain recommendations, compared to 25% (n=8) of those who did not use the app. At 6 months postpartum, the mean BMI was increased in both groups to 25.6 for users and 25.7 for non-users. The mean difference of BMI among users and non-users was 0.6 and 0.9, respectively, corresponding to an increase of BMI of 2.4 for users and 3.6 for non-users.

Prior to pregnancy, the users stated on average a healthier lifestyle, as measured by physical activity and diet compared to the non-users. At 6 months postpartum, physical activity and healthy diet had decreased in both groups compared to prepregnancy values. A total of 90% (n=20) of the users and all of the non-users stated they wanted to be more physically active, and 75% (n=28) of the users in both groups stated they would like to have a healthier diet. Among the users, 75% (n=20) felt they had been cheerful and in good spirits over the past 4 weeks compared to 25% (n=8) of the non-users, and a larger share of the users than non-users stated that they had control of their own health (90%, n=20 vs 62.5%, n=8). The median household income for users was between DKK 500,000 (US $81,587.5) and DKK 599,999 (US $97,905) and between DKK 400,000 (US $65,270) and DKK 499,999 (US $81,587) for non-users. The median category of education was medium cycle higher education for both users and non-users. All respondents were in a relationship.

**Table 2.** Characteristics of a sample of postpartum women, chosen consecutively in the Region of Southern Denmark between June and December 2020^a^.

Questions	Users (n=20), n (%)	Non-users (n=8), n (%)	All (n=28), n (%)
Age (y)			
20‐29	10 (50)	3 (37.5)	13 (46.4)
30‐39	10 (50)	4 (50)	14 (50)
40+	0 (0)	1 (12.5)	1 (3.6)
Mean age (y)	29.8	32	30.4
Occupation prior to the maternity leave			
Under education/employed	17 (85)	6 (75)	23 (82.1)
Unemployed/other	3 (15)	2 (25)	5 (17.6)
Education level			
Primary/upper secondary education	2 (10)	1 (12.5)	3 (10.7)
Short cycle higher education	4 (20)	1 (12.5)	5 (17.6)
Medium/long cycle higher education	13 (65)	6 (75)	19 (67.9)
Other	1 (5)	0 (0)	1 (3.6)
Yearly household income (DKK)			
Under 300,000 (US $48,952)	4 (20)	1 (12.5)	5 (17.6)
300,000‐499,000 (US $48,952-81,587)	4 (20)	4 (50)	8 (28.6)
500,000 (US $81,587.5) or more	12 (60)	3 (37.5)	15 (53.6)
Number of children in the household			
1 (vs more than 1)	13 (65)	4 (50)	17 (60.7)
Civil status			
Married or in a relationship	20 (100)	8 (100)	28 (100)
Hours spent on screen (phone) per day			
0‐2	11 (55)	3 (37.5)	14 (50)
>2	9 (45)	5 (62.5)	14 (50)
Days of minimum 30-minute physical activity			
0‐2	15 (75)	8 (100)	23 (82.1)
>2	5 (25)	0 (0)	5 (17.6)
Physical activity prior to pregnancy			
0‐2	10 (50)	8 (100)	18 (64.3)
>2 days	10 (50)	0 (0)	10 (35.7)
Would you like to be more physically active?			
Yes	18 (90)	8 (100)	26 (92.9)
Diet prior to pregnancy			
Very healthy/healthy	13 (65)	3 (37.5)	16 (57.1)
Somewhat healthy/very unhealthy/unhealthy	7 (35)	5 (62.5)	12 (42.9)
Diet at present			
Very healthy/healthy	9 (45)	1 (12.5)	10 (35.7)
Somewhat healthy/very unhealthy/unhealthy	11 (55)	7 (87.5)	18 (64.3)
Would you like to eat healthier?			
Yes	15 (75)	6 (75)	21 (75)
Over the past 4 weeks			
I have felt cheerful and in good spirits			
All the time/more than half of the time/some of the time	15 (75)	2 (25)	17 (60.7)
Less than half of the time/at no time	5 (25)	6 (75)	11 (39.3)
Over the past 4 weeks			
I have felt tired			
All the time/more than half of the time/some of the time	8 (40)	4 (50)	12 (42.9)
Less than half of the time/at no time	12 (60)	4 (50)	16 (57.1)
I have control of my health			
Strongly agree/agree	18 (90)	5 (62.5)	23 (82.1)
Neither, disagree, strongly disagree	2 (10)	3 (37.5)	5 (17.6)
What influences my health			
is what I do myself			
Strongly agree/agree	16 (80)	7 (87.5)	23 (82.1)
Neither, disagree, strongly disagree	4 (20)	1 (12.5)	5 (17.6)
BMI prior to pregnancy			
>25 (vs 25 and below)	6 (30)	3 (37.5)	9 (32.1)
Mean score	25	24.8	
Calculated BMI 6 months postpartum			
>25 (vs 25 and below)	6 (30)	4 (50)	10 (35.7)
Mean score	25.6	25.7	
IOM^b^ weight gain recommendations (based on BMI)			
Above	10 (50)	2 (25)	12 (42.9)
Normal	5 (25)	4 (50)	9 (32.1)
Below	5 (25)	2 (25)	7 (25)

a Data collected by a questionnaire, n=28.

b IOM: Institute of Medicine.

The self-reported use of and satisfaction with Healthy Together is displayed in Table 3.

Weekly push messages and podcasts were rated as the most often used content. They were used by 65% (n=20) of the users. About one-third of those who used the module stated that the push notifications motivated them. Half of the respondents stated that they used the exercise videos, while slightly less used the weight tracking. More than half of those who used the module stated it worked well with podcasts and exercise videos. In total, 40% (n=20) of the women tracked their own weight development in the module. Only 15% (n=20) stated they regularly used Healthy Together. The majority stated that Healthy Together was easy to use, and all users found the information in Healthy Together easy to understand. However, more than one-third of the participants answered that they experienced technical issues with the module. When asked if they had any comments, the interface was mentioned as an issue as it was too complicated to log on to the messages, and it seemed stiff and boring. In addition, another platform was recommended by several participants due to technical issues. Only 25% (n=20) of the participants used other apps related to postpartum health. More than half (55%, n=20) stated that Healthy Together had a positive effect on their health status, and 70% (n=20) stated they would recommend it to others.

**Table 3.** Use of the app Healthy Together by a sample of postpartum women chosen consecutively in the Region of Southern Denmark between June and December 2020.^a^

Question and response	N (%)
Which elements have you used in the module?	
Podcasts	13 (65)
Push notifications	13 (65)
Exercise videos	9 (45)
Weight tracking	8 (40)
I have used the module regularly	
Strongly agree	0 (0)
Agree	3 (15)
Neither agree nor disagree	8 (40)
Disagree	5 (25)
Strongly disagree	4 (20)
Don’t know	0 (0)
The module has been easy to use	
Strongly agree	6 (30)
Agree	8 (40)
Neither agree nor disagree	4 (20)
Disagree	1 (5)
Strongly disagree	1 (5)
Don’t know	0 (0)
The information in the module has been easy to understand	
Strongly agree	15 (75)
Agree	5 (25)
Neither agree nor disagree	0 (0)
Disagree	0 (0)
Strongly disagree	0 (0)
Don’t know	0 (0)
The module has had a positive influence on my health	
Strongly agree	1 (5)
Agree	10 (50)
Neither agree nor disagree	8 (40)
Disagree	0 (0)
Strongly disagree	0 (0)
Don’t know	1 (5)
It has worked well with podcasts	
Strongly agree	5 (25)
Agree	7 (35)
Neither agree nor disagree	5 (25)
Disagree	1 (5)
Strongly disagree	0 (0)
Don’t know	2 (10)
It has worked well with exercise videos	
Strongly agree	5 (25)
Agree	6 (30)
Neither agree nor disagree	2 (10)
Disagree	2 (10)
Strongly disagree	1 (5)
Don’t know	4 (20)
The push notifications have motivated me	
Strongly agree	2 (10)
Agree	5 (25)
Neither agree nor disagree	8 (40)
Disagree	1 (5)
Strongly disagree	1 (5)
Don’t know	3 (15)
I would recommend the module to others	
Strongly agree	4 (20)
Agree	10 (50)
Neither agree nor disagree	2 (10)
Disagree	1 (5)
Strongly disagree	0 (0)
Don’t know	3 (15)
Have you experienced technical issues?	
Yes	7 (35)
No	12 (60)
Don’t know	1 (5)
Do you use other apps related to your health?	
Yes	5 (25)
No	15 (75)
Don’t know	0 (0)

a Non-users have been excluded. Data collected by a questionnaire, n=20.

### Semi-Structured Interviews

#### Overview

In total, 18 semi-structured interviews were conducted. When invited for an interview, mothers who did not use or only scarcely used the module, according to their own perception, explained that they lacked time and energy to focus on it. A total of six overall key themes were identified from the STC: (1) information flow, (2) podcasts as a preferred information channel, (3) information from a trustworthy source, (4) technical barriers, (5) user interface, and (6) weight tracking reminders.

#### Key Theme 1: Information Flow

The women generally felt that the push notification reminded them about the module’s presence and contents and were happy to receive reminders about the information in the module, as it gave them time to obtain and comprehend the information. “*The notifications reminded me about the topics in the module (Healthy Together). It made me more aware of my diet and also that I had to remember to exercise the pelvic floor”* (Mother, participant). When asked about the number of push notifications, most preferred to receive more messages during the first weeks, specifically those concerning the intake of water and healthy food. About two-thirds of the women would like to be introduced to the module at the end of their pregnancy, rather than after the baby was born, as they felt they still had time and energy to focus on new information.

#### Key Theme 2: Podcasts—A Preferred Information Channel

The women were happy to receive information through podcasts, as it was more personal than a leaflet, making them a more preferred information channel. As one mother said, “*A leaflet is way more factual…like a list of things I should keep in mind or do….I do not read them…A podcast on the other hand is more empathetic…It is like they are saying ‘I understand how you feel’…and they are nice to listen to…and it is like I am the fly on the wall…”* (Mother, participant). Some women mentioned that podcasts were convenient, as they could listen while having their hands and eyes free. However, not all the mothers used the podcasts, though they still preferred to receive information in this manner. The women who listened to the podcasts would like more of them; for instance, with information for those who had had a cesarean or a complicated delivery, as their bodies were more vulnerable. In addition, some wished for podcasts on mental well-being and solutions such as meditation.

#### Key Theme 3: Information From a Trustworthy Source

The women who used the exercise videos or listened to the podcasts said they gained new information they felt they could rely on regarding postpartum health and lifestyle, as the information was provided by the hospital, and thus from a trustworthy source. One mother said, “*It was as if someone held my hand”* (Mother, participant). The women liked the combination of the chosen information channels (push notifications, videos, and podcasts) and found them appropriate for delivering the provided information.

#### Key Theme 4: Technical Barriers

Several mothers expressed having experienced technical issues. In most cases, they had been logged off the app and did not receive the weekly push notifications. Consequently, they were not reminded about the module and its content. This would happen if they did not update the app or their phone. One participant said, “*I did not receive the messages…I forgot about the app…and then it became an app among the others…”* (Mother, participant). Hence, the technical problems had a negative impact on the women’s use of Healthy Together.

#### Key Theme 5: User Interface

The women were generally positive about the content, though the user interface was an issue. They did not find the application user-friendly and found it a bit boring. For instance, when receiving push notifications, they would be informed that they could log in to see the message, rather than receiving the message directly on the screen. The main recommendation was using another app for Healthy Together. Finally, some mothers preferred a message that popped up with their own name in it. To further motivate usage, a participant said, “*It would make me feel that they are talking to me, rather than to a broad audience in a standard message”* (Mother, participant). Finally, adding an image of each exercise, next to the video, was recommended so the mothers could do the exercises at their own pace once they had already seen the videos. A minor theme was some participants suggested customizing the app so that the mothers could choose the content they wished to use.

#### Key Theme 6: Weight Tracking Reminders

There was great variation among those who liked the weight tracking reminders and those who did not. For some of the women, being asked about their weight elicited negative feelings, while others found it motivating. This may be related to the individual woman’s progress related to her weight loss. One mother stated, “*It was like being slapped in the face…I had not lost weight, and I knew that…and then it made me feel less motivated to use the app*…” (Mother, participant).

### Triangulation

Overall, there is a concurrence in the data from the semi-structured interviews, the questionnaire, and the data retrieved from the app, My Hospital. The women who participated in an interview had all used the module, whereas 70% (n=20) of those who answered the questionnaire (Table 3). When asked if they would recommend Healthy Together to others, 70% of the users in the questionnaire said yes, which is in line with the results from the semi-structured interviews.

In the interviews, when asked about their experience of using Healthy Together, the mothers were in general happy with the content provided. They highlighted technical problems as an issue in both the questionnaire and the interviews. When asked about suggestions for improvements, it was recommended in both the questionnaire and the interviews to use an independent app for Healthy Together, as the user interface was boring and some experienced technical issues.

In the questionnaire, podcasts and push notifications were rated the most used content, followed by exercise videos and weight tracking reminders, which is in line with the results from the interviews and the data retrieved from the app. According to Table 1, which presents the data retrieved from the app, push notifications were used the most, while podcasts and exercise videos were on average used an equal number of times. However, mothers typically engaged with podcasts only once, whereas they tended to revisit exercise videos multiple times.

In the interviews, mothers who already participated in private postpartum exercise classes explained that they, in general, did not use the exercise videos. The weight tracking was used by less than half of the users. Those who did lose weight were happy to track their weight, while those who did not felt demotivated.

A bit more than half of the users in both the interviews and questionnaire stated that the module had a positive impact on their individual health.

## Discussion

### Principal Findings and Comparison With Previous Works

This study demonstrated that the module Healthy Together in My Hospital has the potential to enable change in lifestyle behaviors in terms of diet and exercise, as it provides continuous reminders with trustworthy guidance and motivation to live healthier. The results did, however, also show that technical issues and the interface in My Hospital were considered barriers for use. The findings further suggest that the specific intervention might not target those in most need for information and guidance.

When looking at physical activity and diet, the intervention has been used more by those who were already physically active, had a healthy diet prior to pregnancy, and were in higher self-reported self-control. As both diet and physical activity deteriorated in both groups, the intervention could benefit all users. The results from the questionnaire indicate that Healthy Together cannot be a stand-alone solution when aiming to assist all new mothers, as it was not used by all. A multicomponent intervention may thus be a way forward to reach a broader group of women postpartum [42]. Consequently, Healthy Together could serve as a supplement to the current services provided to the group of new “unproblematic” mothers who are given standard care. Whether the module should rely more on consultations with HCPs should also be considered. Finally, and according to our previous study, partner support has an impact on mothers’ lifestyles. Thus, how to involve them should be considered further in future studies [24,43,44].

The mean BMI for both users and non-users was close to overweight both before and after the intervention period. Less than half of the participants used the weight tracking in the module, which also included weight tracking reminders. The reminders were intended to work as a “nudge” motivating to behavioral change [45]. Unfortunately, the inclusion of weight tracking reminders appeared to be two-sided, as it motivated those who applied the weight tracking component and did lose weight but had the opposite effect on those who put on weight. For the latter group, it also had a negative impact on their overall use of Healthy Together. Some of the women proposed to handle this problem by making the intervention more customized, thereby letting the women freely choose if they would like to receive weight tracking reminders or not and helping avoid pushing users who are not prepared. Our finding is in keeping with Eysenbach [20], who addresses the issue of push messages that are perceived as being too pushy and the fact that they can have a negative impact on the use of an app.

Push notifications referring to the same content can also decrease motivation to use an app, and thus have an impact on adherence or cause some users to uninstall the app [46]. Some said that a way to accommodate this could be by adding more content (podcasts and videos), for instance on mental well-being, while referring less to already presented content. Some mothers recommended making the module more personal by addressing the users by their name in the push notifications. This is relevant to bring forward as there is evidence that messages that include the receiver’s name compared to standard messages can improve uptake and adherence [47]. In addition, the user interface should be improved by using fewer steps to access the push notifications.

Technical problems had a major impact on the mothers’ use of the module. The results underline the importance of testing on a small scale, when using push notifications, as the sender does not receive information on whether a notification has been received or not. This is especially important when communication goes one way, there is no feedback on the reception and interpretation of a message [48]. Moreover, functionality has been found to affect app usage and to have a direct impact on dropouts if people feel annoyed or lack competencies to use the provided tool [20]. My Hospital may be a mature technology for other modules, though for Healthy Together, which uses push notifications, it has proven to be immature, which should be dealt with prior to further testing (pursuant to EU guidelines [49]).

Data from the summary statistics on average visits to the module showed that all content has been used, but to varying degrees and likely not by all participants. By gradually providing the mothers with reminders and information through push notifications, they expressed there was time to obtain and comprehend the information. In addition, the mothers’ general wish was to be introduced to the module prior to giving birth, as then they still had time to take in new information. In line with Hoq, too much information handed out at the same time and lack of time to understand the information can cause information overload [50]. The use of podcasts was also expressed as being a convenient information channel, as the mothers could absorb the information while having their eyes and hands free. The health care system should reconsider the way it communicates and delivers information, for instance, by using podcasts [16]. In line with the summary statistics from the app, the mothers rated podcasts and push notifications as the content they had used the most. The study raises a broader question about how to communicate and deliver information so that it reaches those who need it the most.

### Strengths and Weaknesses

The reliability of the results from the questionnaire may be limited due to the small sample size, and generalizability may be limited due to dropouts [51]. For the same reason, statistical hypothesis testing was not conducted. In contrast, the qualitative data material is comprehensive, which is a strength. After 18 interviews, an impression of information satiety had been reached [52].

To increase the response rate, all of the women who answered the questionnaire participated in the lottery for a 500 DKK (US $81.6) voucher for baby equipment. Using a lottery can motivate people with low motivation to participate [53], which is a strength. The women who participated in the study had to accept their participation. Therefore, they do not represent all who gave birth, but only those who willingly participated. However, it cannot be concluded whether this has caused a bias compared to all women who gave birth, and in which direction. The mothers were all in a relationship, almost all were employed or students, and were well educated.

The study is likely to suffer from selection bias. Women who were not motivated may have declined the offer to participate. In addition, there may be a selection bias with the overrepresentation of non-users among those who did not respond to the questionnaire, as the questionnaire was sent out via the module and not directly to their addresses. Unfortunately, we do not have information on the non-users. Finally, the questionnaire was self-reported, resulting in a possible bias related to under-reporting of weight and hours spent on the screen (phone), while over-reporting lifestyle (diet and exercise) and the use of Healthy Together.

The combined use of a questionnaire, interviews, and summary statistics from the app in a triangulation is a strength, as it provides more comprehensive information with different perspectives. In total, 18 respondents were interviewed, giving a better understanding of the experiences and attitudes. The respondents in the questionnaire and interviews were not completely identical, as those who accepted an invitation for an interview had mostly used Healthy Together, while the respondents in the questionnaire contained a higher share of non-users.

Credibility was ensured by using a semi-structured interview guide with follow-up questions [54]. A semi-structured interview guide was also used to aim to gather similar data across all interviews. The study was conducted in a local setting. However, literature suggests that postpartum women in general lack similar support [8-10]. Thus, transferability to another setting [54] with similar users seems possible. Confirmability was aimed for by using notes and transcripts from all the interviews. In addition, the results were discussed with the co-authors. Regarding dependability, all interviews were conducted online due to COVID-19 restrictions. Authenticity was aimed for by selecting the appropriate people (postpartum women) and presenting their views and deeper understanding of their attitudes from using Healthy Together. The interviews were conducted via phone or Zoom, which may have caused data loss, as behavior and body language could not be observed. There is, however, as shown by Novick, only limited evidence of data loss when conducting phone interviews [55].

Healthy Together was tested at the beginning of the COVID-19 lockdown. Consequently, exercise groups for new mothers were, in most cases, canceled or broadcast online. Thus, the women had even less physical contact with HCPs. This may have had an impact on their use of Healthy Together, but whether this has increased or decreased use and satisfaction is uncertain. Medical or surgical interventions were not considered in the inclusion of the women, and the impact of this is unknown.

Healthy Together was developed in a tax-financed health care system. The module can potentially be adapted and implemented in other health care systems that focus on promoting a healthy lifestyle. While there are some costs related to the development in another setting, the marginal cost of offering the module is close to zero. Therefore, it has potential to be used on a large scale.

### Conclusion

The study demonstrates that the app module Healthy Together is a potentially feasible tool to assist women in improving their lifestyle postpartum. The information flow with weekly reminders on podcasts, videos, and weight tracking gave the mothers time to obtain and comprehend the information and has most likely increased the overall use of the module. Reactions to the reminders of weight tracking were heterogeneous and, in some cases, negative, suggesting that a customized solution should be considered to avoid demotivation.

Though the intervention did not reach all, the intervention is still relevant for those who use it. The module could benefit from a more personalized and customized approach where the users can choose the content that interests them. Finally, the women should be introduced to the module at the end of their pregnancy, as they still have time and energy to obtain new information.

The study demonstrates that it is important to use a platform that is ready for the purpose. In addition, improving the user interface should be considered. Further research with a larger sample is needed in order to measure the effects related to the women’s lifestyles and health statuses.

## Supplementary material

10.2196/69391 Multimedia Appendix 1 Questionnaire.

10.2196/69391 Multimedia Appendix 2 Semi-structured interview guide.

## Abbreviations

BMI: body mass index
HCP: health care professional
IOM: Institute of Medicine
STC: systematic text condensation
