# Supplementary material for: Behavioral Change Intervention to Promote a Healthier Postpartum Lifestyle: Mixed Methods Pilot Study
Source: JMIR Form Res. 2025 Oct 22;9:e69391. doi: 10.2196/69391 (PMC12543209; doi:10.2196/69391)
Supplement: Multimedia Appendix 1 [file formative-v9-e69391-s001.docx]

### Appendix 1 Questionnaire

**Evaluation of your experience using Healthy Together**

**Use of Healthy Together**

The following questions concern your use of Healthy Together

1) Have you used Healthy Together?

Yes

No (those who answer no will be directed to question no. 13.)

2) Which elements in the module have you used?

Podcasts

Weekly push messages

Exercise videos

Weight tracking

Your experience of using Healthy Together

3) I have used the module regularly.

Strongly agree

Agree

Neither agree nor disagree

Disagree

Strongly disagree

Don’t know

4) The module has been easy to use.

Strongly agree

Agree

Neither agree nor disagree

Disagree

Strongly disagree

Don’t know

5) The information in the module has been easy to understand.

Strongly agree

Agree

Neither agree nor disagree

Disagree

Strongly disagree

Don’t know

6) The module has had a positive influence on my health.

Strongly agree

Agree

Neither agree nor disagree

Disagree

Strongly disagree

Don’t know

7) The module has worked well with podcasts.

Strongly agree

Agree

Neither agree nor disagree

Disagree

Strongly disagree

Don’t know

8) The module has worked well with exercise videos.

Strongly agree

Agree

Neither agree nor disagree

Disagree

Strongly disagree

Don’t know

9) The push notifications have motivated me.

Strongly agree

Agree

Neither agree nor disagree

Disagree

Strongly disagree

Don’t know

10) I would recommend the modules to others.

Strongly agree

Agree

Neither agree nor disagree

Disagree

Strongly disagree

Don’t know

11) Have you experienced technical issues?

Yes

No

If yes, please write what kind of technical issues.

12) Do you use other apps related to your health?

Yes

No

If yes, please write which apps you have used.

**Something about you**

13) What was your occupation prior to going on maternity leave?

Undergoing education

Employed

Unemployed

Other

14) What is the highest education level you have completed?

Primary education

Upper secondary education

Vocational education and training

Short cycle higher education

Medium cycle higher education

Long cycle higher education

PhD

Other

15) What is your household’s yearly income before tax?

Under 100,000

100,000 – 199,999

200,000 – 299,999

300,000 – 399,999

400,000 – 499,999

500,000 – 599,999

600,000 – 699,999

700,000 – 799,999

800,000 or more

16) How many children live in your household?

1

2

3

More than 4

17) Your civil status

Married or in a relationship

Single

Widow

18) How many hours have you spent on your screen (phone) per day, on average, over the past four weeks?

Less than an hour

1- 2 hours

2- 3 hours

3- 4 hours

4- 5 hours

More than 5 hours

19) What is your height? (cm)

20) What is your weight now? (Kg)

21) What was your weight prior to the pregnancy? (kg)

22) How many kg did you gain in your pregnancy? (kg)

23) In a typical week, on how many days prior to your pregnancy did you do moderate intensity sports for at least 30 minutes?

No days

1- 2 days

2- 3 days

3- 4 days

5-7 days

24) In a typical week, on how many days do you do moderate intensity sports for at least 30 minutes?

No days

1- 2 days

2- 3 days

3- 4 days

5-7 days

25) Would you like to be more physically active?

Yes

No

26) How would you describe your diet prior to your pregnancy?

Very healthy

Healthy

Somewhat healthy

Unhealthy

Very unhealthy

27) How would you describe your present diet?

Very healthy

Healthy

Somewhat healthy

Unhealthy

Very unhealthy

28) Would you like to eat healthier than you do now?

Yes

No

29) Over the last four weeks, I have felt cheerful and in good spirits.

All the time

Most of the time

More than half of the time

Some of the time

Less than half of the time

At no time

30) Over the last four weeks, I have felt tired.

All the time

Most of the time

More than half of the time

Some of the time

Less than half of the time

At no time

31) I have control of my own health.

Strongly agree

Agree

Neither agree nor disagree

Disagree

Strongly disagree

Don’t know

32) What influences my health is what I do myself.

Strongly agree

Agree

Neither agree nor disagree

Disagree

Strongly disagree

Don’t know

33) If you have any suggestions for improvements or comments related to Healthy Together, please write them here.
