# Supplementary material for: Behavioral Change Intervention to Promote a Healthier Postpartum Lifestyle: Mixed Methods Pilot Study
Source: JMIR Form Res. 2025 Oct 22;9:e69391. doi: 10.2196/69391 (PMC12543209; doi:10.2196/69391)
Supplement: Multimedia Appendix 2 [file formative-v9-e69391-s002.docx]

### Appendix 2 Semi-structured interview guide

Table S1 Semi-structured interview guide

| Questions | |
| --- | --- |
| Have you used Healthy Together? | (If no, any specific reasons?) |
| What was your experience of using Healthy Together? |  |
|  | (What worked well/not so well?) |
|  | Weight tracking |
|  | Podcasts |
|  | Exercise videos |
|  | Push notifications |
| Do you feel the module has had any impact on your health? | Behavior? Provide an example. |
| Do you have any suggestions for improvements? |  |
| Would you recommend Healthy Together to |  |
| others? |  |
